# Secular trends in HbA1c levels among children and adolescents with type 1 diabetes

**DOI:** 10.3389/fendo.2026.1786897

**Published:** 2026-05-28

**Authors:** Jung Chou, Chao-Hsu Lin, Shih-Kai Kevin Lin, Yann-Jinn Lee, Chi-Yu Huang, Yu-En Kao, Jia-Cian Yu, Wei-Hsin Ting

**Affiliations:** 1 Department of Pediatric Endocrinology, MacKay Children’s Hospital, Taipei, Taiwan; 2 Department of Pediatric Endocrinology, Hsinchu MacKay Memorial Hospital, Hsinchu, Taiwan; 3 Department of Biological Science and Technology, National Chiao-Tung University, Hsinchu, Taiwan; 4 Department of Public Health, Institute of Epidemiology and Preventive Medicine, College of Public Health, National Taiwan University, Taipei, Taiwan; 5 Department of Medicine, MacKay Medical College, New Taipei City, Taiwan; 6 Department of Medical Research, Tamsui MacKay Memorial Hospital, New Taipei City, Taiwan

**Keywords:** glycemic control, HbA1c, linear mixed-effects models, pediatric, secular trends, type 1 diabetes

## Abstract

**Introduction:**

The management of type 1 diabetes (T1D) has significantly improved over the past two decades in parallel with advancements such as medical nutrition therapy, long-acting and rapid-acting insulin analogues, reimbursement for blood glucose test strips for T1D in Taiwan, and the widespread use of continuous glucose monitoring (CGM). This study aimed to evaluate whether glycemic control has improved among children and adolescents with T1D at MacKay Children’s Hospital and Hsinchu MacKay Memorial Hospital from January 1, 2000, to December 31, 2023, and to investigate whether trends varied by sociodemographic factors.

**Materials and methods:**

We analyzed electronic medical records of 688 patients with T1D, registered between 1st, Jan, 2000 and 31th, Dec, 2023 from MacKay Children’s Hospital and Hsinchu MacKay Children’s Hospital, which contained repeated measures of HbA1c. HbA1c levels and achievement rate of optimal HbA1c level, defined as HbA1c <7.0%, were treated as outcome variables for analysis. A linear mixed-effects model (LME) with a random intercept per patient was used as the primary analytic approach to estimate the calendar-year trend in HbA1c, adjusting for diabetes duration at each visit, sex, DKA status, and age group at diagnosis. Formal interaction terms between calendar year and each subgroup variable were tested using likelihood ratio tests (LRT). For the binary outcome of HbA1c <7.0%, a generalized estimating equations (GEE) approach with an exchangeable correlation structure was applied. All analyses were performed using R software (version 4.3.2).

**Results:**

In the LME, HbA1c declined significantly over the study period after adjusting for diabetes duration and patient characteristics (β = −0.037% per year, 95% CI −0.053 to −0.021, p < 0.001), corresponding to an estimated overall decrease of approximately 0.84% over 23 years. The annual mean HbA1c decreased from 9.17% in 2000 to 7.99% in 2023. Formal interaction testing revealed that the rate of HbA1c improvement differed significantly by sex (LRT p = 0.002), DKA status at presentation (LRT p < 0.001), and age group at diagnosis (LRT p < 0.001). Female patients and those without DKA at presentation showed more pronounced calendar-year improvements. Adolescents diagnosed at 12–18 years consistently exhibited the highest HbA1c levels (β = +0.790% vs. 0–6 yr group, p < 0.001), but also showed the steepest rate of improvement (β = −0.084%/yr). The proportion of HbA1c measurements below 7.0% showed a statistically significant increase per GEE analysis (OR = 1.029 per year, 95% CI 1.011–1.047, p = 0.002), although the annual achievement rate trend did not reach significance by Kendall’s tau (τ = 0.16, p = 0.29).

**Conclusions:**

HbA1c levels among children and adolescents with T1D showed a statistically significant declining trend over 23 years at two centers in Taiwan, in parallel with the progressive adoption of diabetes technology and evolving NHI policy. The rate of improvement differed significantly across subgroups defined by sex, DKA status at presentation, and age group at diagnosis. Despite overall improvement, the 12–18 year age group consistently maintained the highest HbA1c levels throughout follow-up, and the youngest (0–6 year) group showed no significant secular improvement, highlighting persistent clinical challenges across developmental stages.

## Introduction

Type 1 diabetes (T1D) is associated with significant reduction in life expectancy and is a leading cause of complications such as end-stage renal disease, retinopathy, and neuropathy (1). Glycemic control is primarily assessed through glycated hemoglobin (HbA1c) levels. Evidence indicates that achieving lower HbA1c levels can substantially reduce the incidence of these complications and improve overall survival (2, 3). To optimize outcomes, the International Society for Pediatric and Adolescent Diabetes (ISPAD) and the American Diabetes Association (ADA) recommend an HbA1c target of <53 mmol/mol (<7.0%) for children, adolescents, and most non-pregnant adults. Stricter targets of <48 mmol/mol (<6.5%) may be appropriate for certain adults if they can be achieved without significant hypoglycemia (4, 5). Conversely, less stringent targets are recommended for individuals with frequent severe hypoglycemia, significant comorbidities, or limited life expectancy (4, 5).

Over the past two decades, the management of T1D in Taiwan has evolved substantially. Progressive National Health Insurance (NHI) policy expansions—including reimbursement for self-monitoring blood glucose strips in 2010, continuous glucose monitoring (CGM) in 2017, and newer long-acting insulin analogues from 2016 onward—alongside the increasing clinical adoption of insulin pumps, may have contributed to changes in glycemic outcomes in this population. However, because these developments occurred concurrently and were not individually randomized, their specific contributions to HbA1c trends cannot be formally isolated in an observational study. The present study describes population-level trends in glycemic control over time, while acknowledging these evolving contextual factors as plausible explanatory contributors.

Declining secular trends in HbA1c have been documented in multiple high-income countries. Mair et al. studied T1D in Scotland between 2004 and 2016 and reported a decrease in median HbA1c from 8.7% to 8.4%, with the most notable reductions observed in children and adolescents (6). Haynes et al. found that between 1995 and 2016, the annual mean HbA1c decreased from 8.3% to 7.8% in the DPV cohort and from 9.2% to 8.3% in the WACDD cohort (7). Alissa et al. reported that the mean HbA1c for the entire cohort was 10.56% at the beginning of the study period in 2016 and decreased by 0.67% to a mean of 9.89% in 2019 (8). A similar trend was observed in a study conducted between 2013 and 2022, in which the mean HbA1c decreased from 8.2% to 7.6% (9).

In the literature, female patients with T1D consistently exhibit higher HbA1c levels than male patients, with a more pronounced deterioration during puberty. Evidence from large multinational registries and systematic reviews has consistently demonstrated that this sex-related disparity is already present at diagnosis, persists throughout the course of treatment, and reaches its peak during adolescence (10–14). Additionally, the adolescent age group consistently exhibited higher HbA1c levels compared to the younger child age group (6, 7).

The mean rate of optimal glycemic control (HbA1c <7.0%) among children and adolescents varies considerably across countries, reflecting the multifactorial influences of healthcare systems, access to diabetes technology, and socioeconomic conditions. In one study spanning from 2013 to 2022, the rate of achieving HbA1c targets of <7% increased substantially, rising from 19.0% to 38.8%, a trend that paralleled the increasing adoption of diabetes technologies (9). Though differences existed across registries, the highest rates of achieving optimal glycemic control were reported in Swediabkids (57.1%), ČENDA in the Czech Republic (53.6%), and Denmark (48.4%), whereas registries from Australia (ADDN), the United States (T1DX-QI), and the United Kingdom (NPDA) reported considerably lower rates, around 23–26% (9). Even lower rates were observed in low-income countries (15).

In Asia, longitudinal data on HbA1c trends in children and adolescents with T1D remain limited, and the available evidence presents a heterogeneous picture. Park et al. analyzed temporal trends in glycemic control among 752 Korean children and adolescents with T1D between 2010 and 2019, and reported a significant decrease in mean HbA1c from 8.56% to 8.01%, concurrent with a marked increase in CGM and insulin pump use (16). In Japan, the JSGIT multicenter cohort documented a significant decrease in HbA1c levels between 1995 and 2013, accompanied by a concurrent reduction in severe hypoglycemic events and an increase in the use of bolus insulin analogue regimens (17). Data from east China reported a mean HbA1c of 8.1% ± 2.2% among children with diabetes in 2010–2020, with internal migrant children exhibiting significantly poorer glycemic control than resident patients (18). These Asian studies share a common finding of substantial room for improvement, and highlight that glycemic outcomes in this region are influenced by access to diabetes technology and healthcare policy—contextual factors that differ markedly from Western registry settings. To our knowledge, no comparable long-term longitudinal analysis of HbA1c secular trends exists for pediatric T1D in Taiwan.

Epidemiological data indicates that the incidence of type 1 diabetes mellitus (T1DM) in Taiwanese pediatric populations remains substantially lower than that observed in Western countries. Between 2003 and 2008, the incidence was estimated at 5.3 cases per 100,000 children aged 0–14 years (19). A subsequent longitudinal analysis encompassing individuals under 20 years of age demonstrated an incidence of 6.30 per 100,000 person-years in 2001, which slightly decreased to 5.02 per 100,000 person-years by 2015 (20). This epidemiological profile contrasts sharply with Western cohorts; notably, Finland reports the highest global incidence at approximately 57 cases per 100,000 children, while other high-income nations, including the United States, Canada, and Australia, report rates exceeding 20 per 100,000 (21). Overall, the incidence of T1DM in Taiwan aligns with the broader trends observed across Asian populations, which consistently exhibit a lower disease burden relative to European and North American cohorts (22).

Therefore, this study had two primary aims: (1) to describe the secular trend in HbA1c levels among children and adolescents with T1D managed at two MacKay-affiliated centers in Taiwan over a 23-year period (2000–2023), and (2) to evaluate whether the calendar-year trend in HbA1c differed across subgroups defined by sex, DKA status at presentation, and age group at diagnosis. These analyses were conducted using a linear mixed-effects modeling framework that accounts for the repeated-measures structure of the data and adjusts for diabetes duration, in addition to baseline patient characteristics.

## Materials and methods

### Study population

We retrospectively collected data on patients diagnosed with T1D between January 1, 2000, and December 31, 2023, at ≤18 years of age, at MacKay Children’s Hospital (Taipei) and Hsinchu MacKay Memorial Hospital (Hsinchu). Patients were excluded if they had fewer than five HbA1c measurements (to ensure adequate longitudinal information) or if data on DKA status at initial presentation were unavailable. Excluded patients did not differ significantly from included patients in age at diagnosis (8.9 ± 4.2 vs. 8.7 ± 4.2 years, p = 0.619), sex distribution (male 48.4% vs. 46.5%, p = 0.827), or DKA rate, supporting the representativeness of the analytic sample. To assess robustness, we refitted the primary model across five inclusion thresholds (≥1 to ≥5 visits); results are reported in **Supplementary Table 1**.

### Diagnostic era classification

Patients were classified into three diagnostic eras reflecting key transitions in the NHI reimbursement landscape for T1D management in Taiwan. Era 1 (2000–2007) represents the period prior to NHI coverage of SMBG test strips. Era 2 (2008–2015) encompasses the period following the 2010 introduction of NHI reimbursement for SMBG test strips (120 strips per month), which substantially enhanced monitoring access. Era 3 (2016–2023) corresponds to the period in which CGM was first covered by NHI (from March 2017) and newer long-acting insulin analogues—including insulin glargine U300 (2016) and insulin degludec (2017)—became available under NHI reimbursement.

### Measurements of HbA1c and other variables

HbA1c levels were measured using various clinical methods, all standardized to the assay employed in the Diabetes Control and Complications Trial (DCCT), and recorded in percentage units. Over the 23-year observation period, the primary HbA1c assay platform used at both institutions remained consistent (HPLC-based ion-exchange chromatography), with periodic calibration to NGSP/IFCC standards; no systematic laboratory drift was identified. Optimal glycemic control was defined as an HbA1c level of <7.0% for individuals aged ≤18 years, in accordance with the American Diabetes Association (ADA) guidelines (5).

Clinical variables recorded for each patient included age at T1D diagnosis, gender, date of T1D diagnosis, date of birth, and the presence of DKA at initial presentation. Diabetes duration at each HbA1c measurement visit was computed as the time from diagnosis date to visit date, in years. Calendar year of each visit was extracted from the date of visit. Additionally, we collected data on the adoption of CGM from 2020 to 2023 and the proportion of patients using long-acting insulin analogues from 2018 to 2023.

### Case definition

T1D was defined based on the criteria set by the ADA (23) with the presence of one or more of the following autoimmune markers: islet cell autoantibodies, autoantibodies to glutamic acid decarboxylase (GADA), insulinoma-associated protein 2 (IA2A), zinc transporter 8 antibody (ZnT8A) or insulin (24, 25). Patients without evidence of β-cell autoimmunity were diagnosed based on serum C-peptide of <0.7 mmol/l (2.1 ng/ml) at random or <1.1 mmol/l (3.3 ng/ml) 6 minutes post glucagon stimulation test (26). DKA was defined as glucose ≥ 200 mg/dL, PH ≤ 7.3 or HCO3 ≤ 15 mmHg, and a positive ketone (either in serum or urine) (27).

### Statistical analysis

All statistical analyses were performed using R software (version 4.3.2; R Foundation for Statistical Computing, Vienna, Austria), with a two-sided P-value of <0.05 considered statistically significant.

Primary analysis. A linear mixed-effects model (LME) was used as the primary analytic approach to characterize the secular trend in HbA1c and account for the repeated-measures structure of the data. Each patient contributed a random intercept, allowing for between-patient heterogeneity in baseline HbA1c level. Fixed effects included: calendar year (continuous, centered at 2011), diabetes duration at each visit (time-varying, in years), sex, DKA status at initial presentation, and age group at diagnosis (0–6, 6–12, or 12–18 years). Calendar year and diabetes duration were entered as separate time-related predictors to disentangle secular trends from within-patient disease progression effects. Parameters were estimated using restricted maximum likelihood (REML). Variance components (between-patient variance τ², residual variance σ², and intraclass correlation coefficient [ICC]) are reported alongside fixed-effect estimates with 95% confidence intervals.

Subgroup trend analyses and interaction testing. To evaluate whether the calendar-year trend in HbA1c differed by subgroup, formal interaction terms between calendar year and each subgroup variable (sex, DKA status, age group at diagnosis) were tested using likelihood ratio tests (LRT), with models refitted under maximum likelihood (REML = FALSE). A significant interaction indicates that the rate of HbA1c change over time differed between subgroups beyond chance. Stratified LME models were additionally fitted within each subgroup to estimate subgroup-specific calendar-year slopes with confidence intervals.

Binary outcome analysis. The proportion of HbA1c measurements falling below 7.0% was analyzed using generalized estimating equations (GEE) with a binomial family and exchangeable working correlation structure. Results are expressed as odds ratios (OR) with 95% confidence intervals. The annual trend in achievement rate was additionally evaluated using Kendall’s tau rank correlation applied to annual aggregate proportions.

Descriptive and supplementary analyses. Annual means and medians of HbA1c were computed and visualized using box plots with superimposed mean trend lines. The Jonckheere–Terpstra trend test was used as a supplementary nonparametric evaluation of ordered differences in HbA1c across calendar years applied to annual aggregate means.

Selection bias assessment. Patients excluded due to having fewer than five HbA1c measurements were compared to included patients on key baseline characteristics using independent-samples t-tests and chi-square tests.

## Results

### Study population

**Figure 1:**
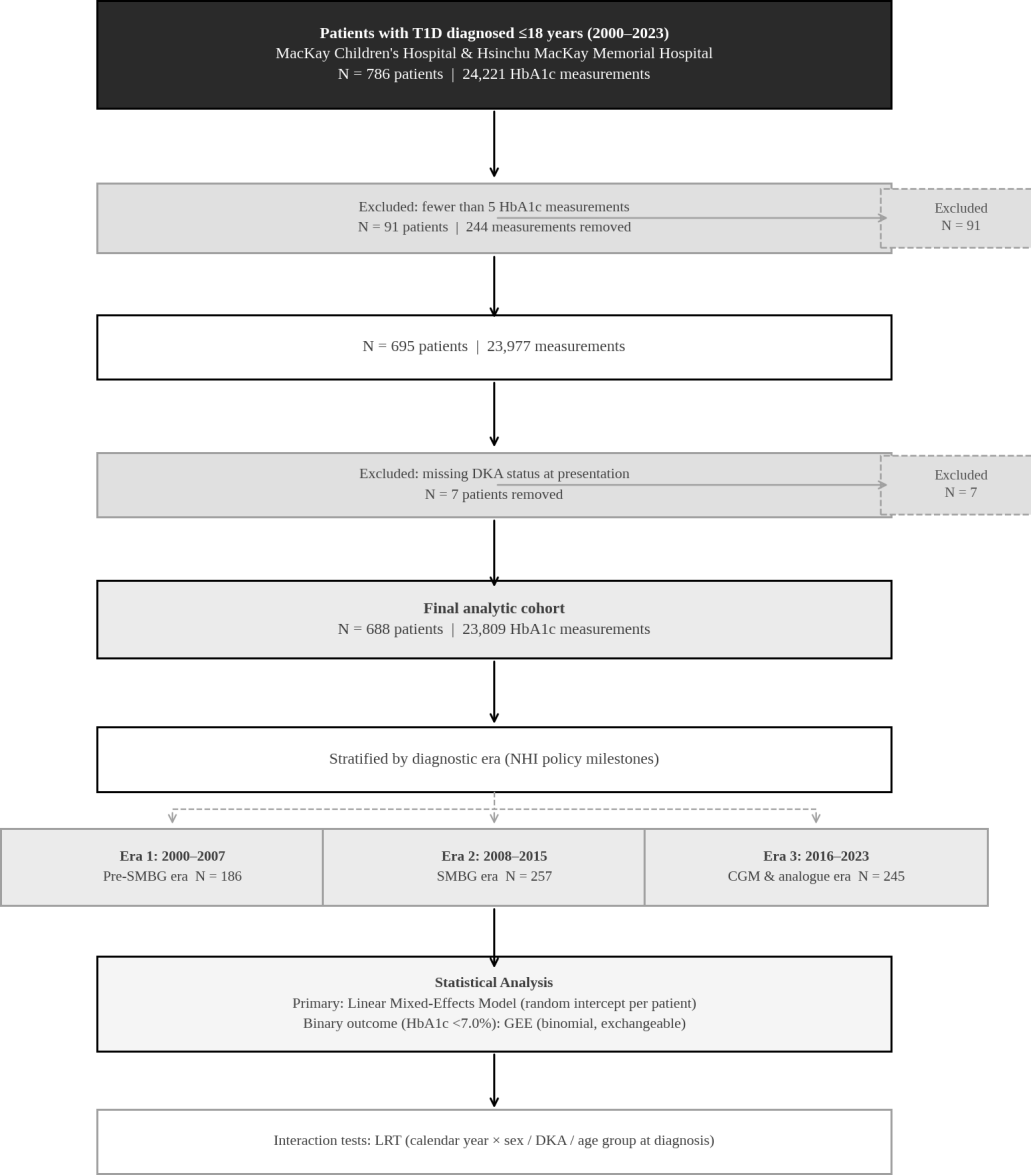
Patient enrollment flow chart. Of 786 patients with T1D initially identified, 91 were excluded for having fewer than five HbA1c measurements and 7 for missing DKA data, yielding a final analytic cohort of 688 patients with 23,809 measurements.

Between January 1, 2000, and December 31, 2023, 786 patients with T1D aged ≤18 years were initially identified. After excluding 91 patients with fewer than five HbA1c measurements and 7 patients with missing DKA data, the final analytic cohort comprised 688 patients contributing 23,809 HbA1c measurements (**Figure 1**). The median number of measurements per patient was 30 (IQR 16–51), reflecting the long-term nature of follow-up. Excluded patients did not differ significantly from included patients in age at diagnosis, sex, or DKA status (all p > 0.6), supporting the validity of the analytic sample. Sensitivity analyses across inclusion thresholds (≥1 to ≥5 visits) confirmed that the primary calendar-year estimate was consistent and statistically significant across all thresholds ≥2 visits (β range: −0.026 to −0.037%/yr; all p ≤ 0.001; **Supplementary Table 1**).

The cohort was stratified into three diagnostic eras: 186 patients in Era 1 (2000–2007), 257 in Era 2 (2008–2015), and 245 in Era 3 (2016–2023). No significant differences were observed across eras in sex distribution (p = 0.386), age group at diagnosis (p = 0.163), or DKA rate at presentation (p = 0.235). Mean age at diagnosis was slightly younger in Era 1 (7.9 ± 4.0 years) compared to Era 2 and 3 (8.8–8.9 ± 4.3–4.4 years; p = 0.041). Follow-up duration was longer in earlier eras by design (Era 1: 13.9 ± 5.5 years; Era 3: 4.2 ± 2.1 years; p < 0.001). Mean first HbA1c did not differ significantly across eras (11.4–11.8%; p = 0.438), indicating comparable glycemic status at diagnosis (**Table 1**).

**Table 1:** Demographic and clinical characteristics of 688 children and adolescents with T1D, stratified by diagnostic era.

Clinical characteristics	2000–2007 N = 186	2008–2015 N = 257	2016–2023 N = 245	P-value
Age group at diagnosis (n, %)				
0–6 years	64 (34.4)	71 (27.6)	74 (31.5)	0.163
6–12 years	86 (46.2)	110 (42.8)	104 (42.4)	
12–18 years	36 (19.4)	76 (29.6)	67 (27.4)	
Gender (n, %)				
Female	99 (53.2)	146 (56.8)	124 (50.6)	0.386
Male	87 (46.8)	111 (43.2)	121 (49.4)	
DKA at diagnosis (n, %)				
Yes	113 (60.8)	137 (53.3)	139 (56.7)	0.235
No	73 (39.2)	120 (46.7)	106 (43.3)	
Age at diagnosis, years (mean ± SD)				
Age at diagnosis	7.9 ± 4.0	8.8 ± 4.3	8.9 ± 4.4	0.041
Follow-up duration, years (mean ± SD)				
Follow-up duration	13.9 ± 5.5	9.3 ± 4.6	4.2 ± 2.1	<0.001
HbA1c measurements per patient (median, IQR)				
Measurements/patient	55 (36–73)	35 (19–53)	14 (8–22)	<0.001
First HbA1c, % (mean ± SD)				
First HbA1c	11.4 ± 3.4	11.6 ± 3.2	11.8 ± 3.1	0.438

Values are n (%) unless otherwise stated. Era 1 = 2000–2007 (prior to SMBG reimbursement); Era 2 = 2008–2015 (SMBG era); Era 3 = 2016–2023 (CGM and newer insulin analogue era). Follow-up duration and measurements per patient are longer in earlier eras by design, as patients diagnosed earlier had more time to accumulate visits by the study end date. P-values from chi-square tests (categorical) or one-way ANOVA (continuous). SMBG, self-monitoring of blood glucose; CGM, continuous glucose monitoring.

### Secular trends in HbA1c: primary LME analysis

**Figure 2:**
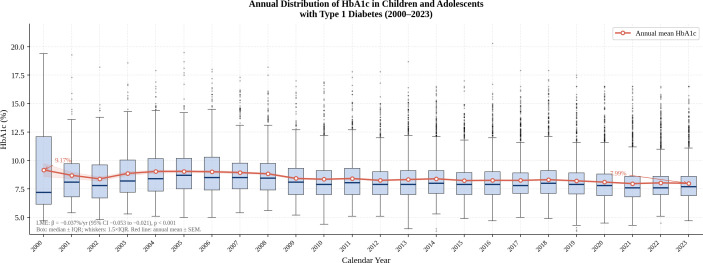
Annual distribution of HbA1c levels in children and adolescents with type 1 diabetes (2000–2023). Box plots display the median (horizontal line), interquartile range (box), and 1.5× IQR whiskers for each calendar year. The red line with circles indicates the annual mean HbA1c. Mean HbA1c declined from 9.17% in 2000 to 7.99% in 2023 (LME: β = −0.037%/yr, 95% CI −0.053 to −0.021, p < 0.001).

In the primary LME analysis, HbA1c declined significantly over the study period after adjusting for diabetes duration at each visit, sex, DKA status, and age group (β = −0.037% per year, 95% CI −0.053 to −0.021, p < 0.001; **Table 2**). This corresponds to an estimated adjusted decrease of approximately 0.84% over the 23-year observation period. The annual mean HbA1c decreased from 9.17% in 2000 to 7.99% in 2023 (**Figure 2**). The between-patient variance was τ² = 1.826 and the residual variance σ² = 1.929, yielding an ICC of 0.486, confirming the appropriateness of the random-intercept structure.

**Table 2:** Linear mixed-effects model: fixed and random effects.

Variable	β (95% CI)	SE	z	p-value	
Fixed Effects					
Calendar year (per year, centered at 2011)	−0.037 (−0.053, −0.021)	0.008	−4.61	<0.001	***
Diabetes duration at visit (per year)	−0.009 (−0.025, 0.006)	0.008	−1.17	0.242	
Male (vs. Female)	−0.055 (−0.263, 0.153)	0.106	−0.52	0.605	
DKA at presentation (vs. No DKA)	+0.207 (−0.005, 0.420)	0.108	+1.91	0.056	
Age group 6–12 yr at diagnosis (vs. 0–6 yr)	+0.241 (−0.004, 0.485)	0.125	+1.93	0.054	
Age group 12–18 yr at diagnosis (vs. 0–6 yr)	+0.790 (0.507, 1.074)	0.144	+5.46	<0.001	***
Random Effects					
Between-patient variance (τ²)	1.826				
Residual variance (σ²)	1.929				
Intraclass correlation coefficient (ICC)	0.486				

β, regression coefficient; CI, confidence interval; SE, standard error. Calendar year centered at 2011 (midpoint of the study period). Diabetes duration entered as a continuous variable (mean-centered). Age group at diagnosis entered as a categorical variable (reference category: 0–6 yr at diagnosis). Reference categories for other variables: Female, No DKA at presentation. *** p < 0.001.

Outcome variable: HbA1c (%). N = 23,809 observations; N = 688 patients. Model: random intercept per patient; REML estimation.

Consistent with prior literature, the 12–18 year age group at diagnosis was independently associated with significantly higher HbA1c levels compared to the 0–6 year group (β = +0.790%, 95% CI 0.507–1.074, p < 0.001). Regarding secondary predictors, both the 6–12 year age group at diagnosis (β = +0.241%, p = 0.054) and the presence of DKA at presentation (β = +0.207%, p = 0.056) exhibited borderline associations with higher mean HbA1c levels, although these did not reach formal statistical significance.

### Subgroup analyses and interaction testing

**Figure 3:**
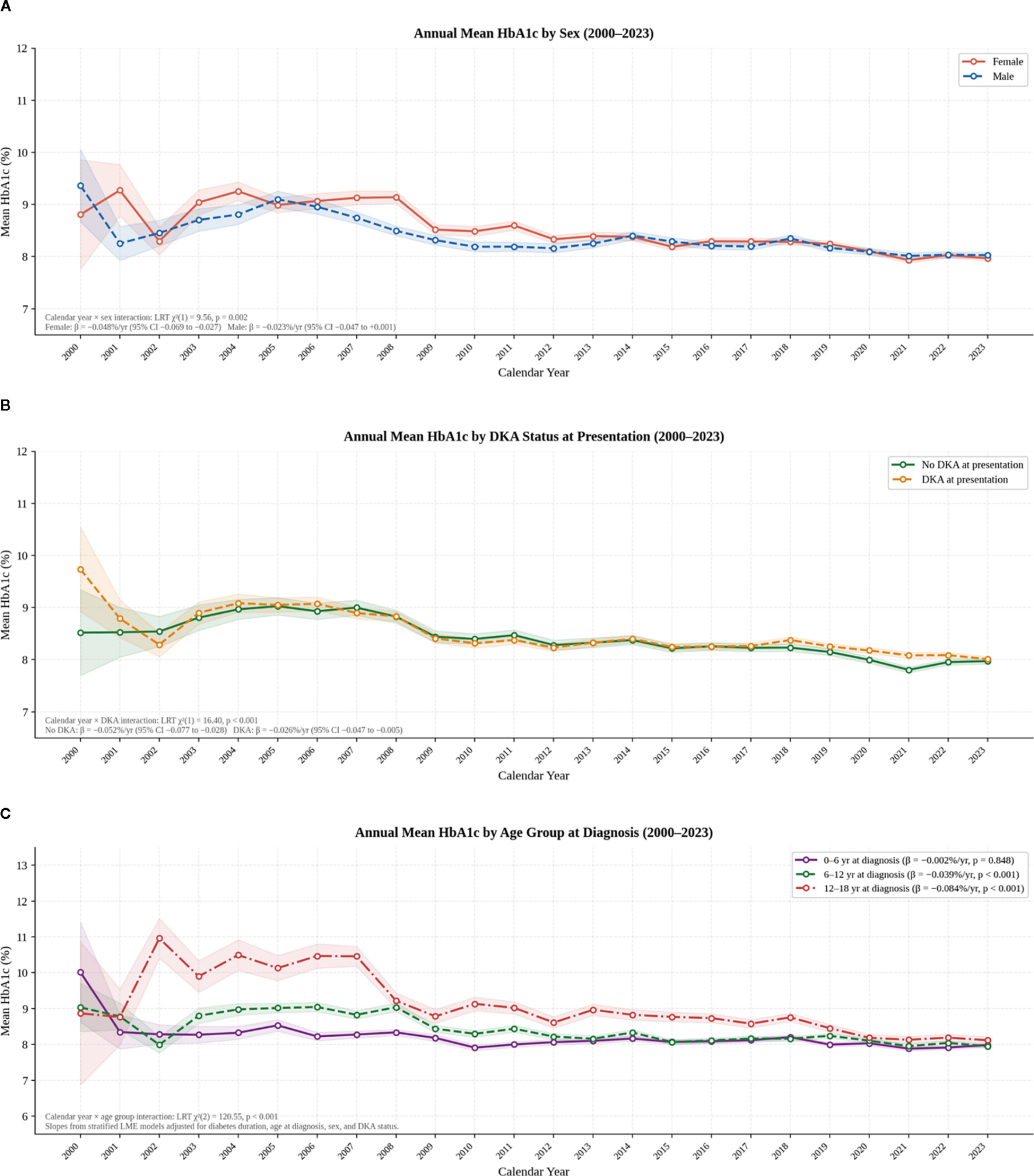
**(A)** Annual mean HbA1c by sex (2000-2023). Lines represent annual mean HbA1c for female (red) and male (blue) patients. Shaded bands represent ±1 SEM. The calendar-year × sex interaction was statistically significant (LRT χ²(1) = 9.56, p = 0.002), indicating that females improved more rapidly (β = −0.048%/yr) than males (β = −0.023%/yr). **(B)** Annual mean HbA1c by DKA status at presentation (2000–2023). Lines represent annual mean HbA1c for patients without DKA (green) and with DKA (orange) at initial presentation. The calendar-year × DKA interaction was statistically significant (LRT χ²(1) = 16.40, p < 0.001). Patients without DKA improved more rapidly (β = −0.052%/yr) than those with DKA (β = −0.026%/yr). **(C)** Annual mean HbA1c by age group at diagnosis (2000–2023). Lines represent annual mean HbA1c for patients diagnosed at 0–6 years (purple), 6–12 years (teal), and 12–18 years (red). The calendar-year × age group interaction was highly significant (LRT χ²(2) = 120.55, p < 0.001). The 12–18 year group showed the steepest improvement (β = −0.084%/yr), the 6–12 year group showed intermediate improvement (β = −0.039%/yr), and the 0–6 year group showed no significant calendar-year trend (β = −0.002%/yr, p = 0.848).

Formal interaction testing revealed that the calendar-year trend in HbA1c differed significantly by all three subgroup variables (**Table 3**; **Figures 3A–C**).

**Table 3:** Subgroup-specific calendar-year slopes and formal interaction tests.

Subgroup	β/yr female or No-DKA or 0–6 yr (95% CI)	β/yr male or DKA or other (95% CI)	LRT χ² (df)	Interaction p-value
Sex	Female: −0.048 (−0.069, −0.027)	Male: −0.023 (−0.047, +0.001)	9.56 (df=1)	0.002
DKA status at presentation	No DKA: −0.052 (−0.077, −0.028)	DKA: −0.026 (−0.047, −0.005)	16.40 (df=1)	<0.001
Age group at diagnosis	0–6 yr: −0.002 (−0.024, +0.019)	6–12 yr: −0.039 (−0.060, −0.018) 12–18 yr: −0.084 (−0.130, −0.038)	120.55 (df=2)	<0.001

β/yr, estimated change in HbA1c per calendar year from stratified LME models with random intercept per patient, adjusted for diabetes duration, age group at diagnosis, sex and DKA status. LRT, likelihood ratio test comparing models with and without the interaction term (REML = FALSE). The interaction p-value tests whether the calendar-year slope differs significantly between subgroups.

Sex. The LRT for the calendar year × sex interaction was statistically significant (χ²(1) = 9.56, p = 0.002). In stratified analyses, female patients showed a more pronounced calendar-year improvement (β = −0.048%/yr, 95% CI −0.069 to −0.027, p < 0.001) compared to male patients (β = −0.023%/yr, 95% CI −0.047 to +0.001, p = 0.056). This finding indicates that over the 23-year period, glycemic control improved more rapidly among females than males.

DKA status. The LRT for the calendar year × DKA interaction was also significant (χ²(1) = 16.40, p < 0.001). Patients without DKA at presentation showed a steeper calendar-year decline in HbA1c (β = −0.052%/yr, 95% CI −0.077 to −0.028, p < 0.001) than those with DKA (β = −0.026%/yr, 95% CI −0.047 to −0.005, p = 0.014).

Age group at diagnosis. The interaction between calendar year and age group at diagnosis was highly significant (χ²(2) = 120.55, p < 0.001). Stratified analyses revealed divergent trajectories: the 0–6 year group showed no significant calendar-year improvement (β = −0.002%/yr, p = 0.848), whereas the 6–12 year group (β = −0.039%/yr, p < 0.001) and the 12–18 year group (β = −0.084%/yr, p < 0.001) demonstrated significant and progressively steeper improvements. Thus, although adolescents diagnosed at 12–18 years continued to have the highest absolute HbA1c levels throughout follow-up, they also showed the greatest magnitude of secular improvement.

### Achievement of optimal glycemic control (HbA1c <7.0%)

**Figure 4:**
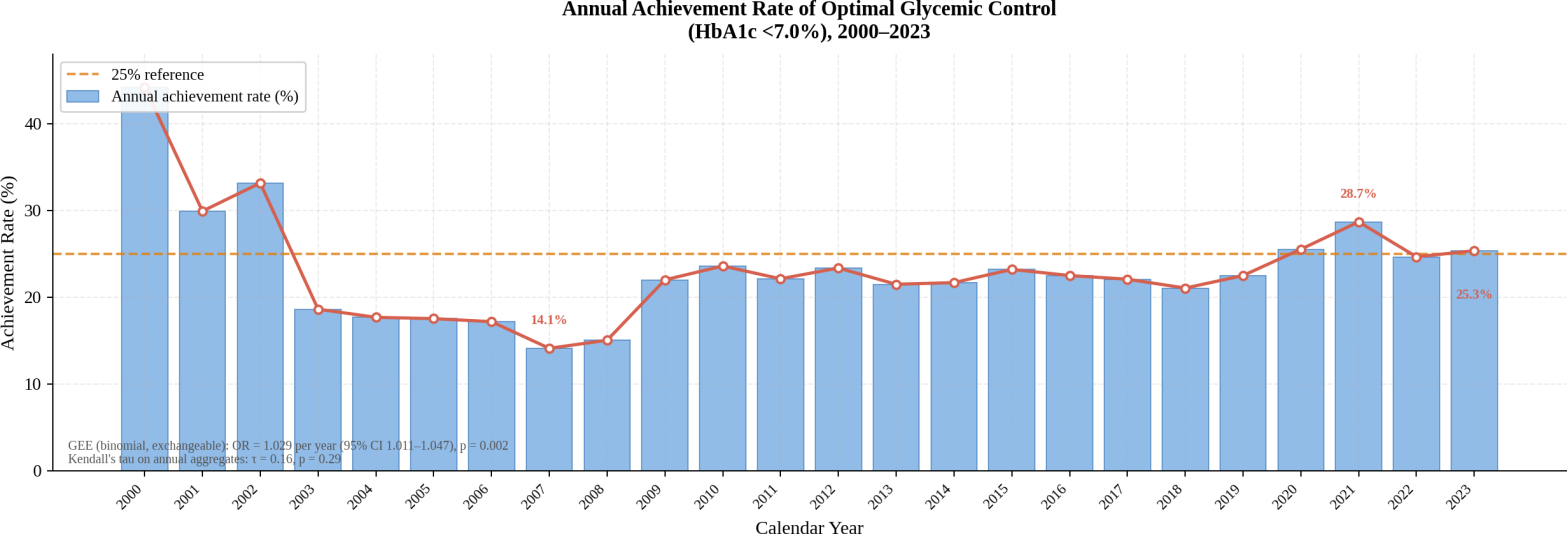
Annual achievement rate of optimal HbA1c (<7.0%) from 2000 to 2023. Bars and the superimposed line indicate the annual proportion of HbA1c measurements (measurement-level) below 7.0%. The GEE analysis was conducted at the measurement level, with exchangeable correlation structure accounting for repeated measures within patients; OR = 1.029 per year (95% CI 1.011–1.047, p = 0.002) reflects the change in odds per calendar year that a given HbA1c measurement falls below 7.0%. The annual aggregate trend did not reach statistical significance by Kendall’s tau (τ = 0.16, p = 0.29).

The GEE analysis, conducted at the measurement level (one observation per HbA1c visit), demonstrated a statistically significant increase in the odds of achieving HbA1c <7.0% over the study period (OR = 1.029 per year, 95% CI 1.011–1.047, p = 0.002), indicating that each calendar year was associated with a 2.9% increase in the odds of meeting the glycemic target. Descriptively, the annual achievement rate increased from 14.1% in 2007 to 28.7% in 2021 and remained at 25.3% in 2023 (**Figure 4**). However, the overall trend in annual achievement proportions did not reach statistical significance by the supplementary Kendall’s tau analysis applied to annual aggregates (τ = 0.16, p = 0.29), likely reflecting year-to-year variability in the proportion of patients eligible for measurement in each calendar year.

### Adoption of diabetes technology

At MacKay Children’s Hospital, CGM adoption expanded substantially from 2020 to 2023, with the number of prescribed CGM sensors increasing from 193 in 2020 to 1,208 in 2023. The proportion of patients with T1D using long-acting insulin analogues rose from 14.9% in 2018 to 71.4% in 2023.

## Discussion

In this 23-year longitudinal study of 688 children and adolescents with T1D from two centers in Taiwan, we demonstrate a statistically significant decline in HbA1c levels over time after adjustment for diabetes duration, sex, DKA status, and age group (β = −0.037%/yr, p < 0.001). Importantly, formal interaction testing revealed that the rate of improvement differed significantly by sex, DKA status at presentation, and age group at diagnosis.

The overall declining trend observed in our cohort is consistent with secular improvements reported in Scotland, Australia, Germany/Austria, and multinational registries (6, 8, 9, 28). However, in contrast to registry studies from the United States, which documented a rise in HbA1c from 8.0% to 8.5% between 2010 and 2018 (29), our cohort demonstrates sustained improvement—suggesting that differences in care coordination, technology access, and insurance coverage may contribute to divergent national trends (29).

The most clinically novel finding of our study is the significant interaction between calendar year and subgroup membership. Female patients improved more rapidly than males (β = −0.048 vs. −0.023%/yr; interaction p = 0.002). This pattern may reflect greater responsiveness among females to structured diabetes education, CGM-based feedback, and multidisciplinary care programs. Prior literature has consistently documented higher baseline HbA1c in females during adolescence (7, 11–14, 30). This sex difference may be attributable to multiple factors, including pubertal hormonal changes, increased insulin requirements, psychosocial stressors, weight gain, and self-management behaviors. In addition, adolescent girls are more likely than boys to present with higher BMI, dyslipidemia, diabetic ketoacidosis, and increased risk of hospitalization during this period (10, 12, 30–32). The lack of stratification by pubertal status in our study and other studies may have diluted potential sex-related differences, possibly masking the post-pubertal deterioration in HbA1c levels observed among females (33–35). Similarly, patients without DKA at presentation showed more pronounced calendar-year improvement than those with DKA (β = −0.052 vs. −0.026%/yr; interaction p < 0.001). Patients presenting without DKA may represent a group with earlier diagnosis, closer surveillance, or better health literacy, allowing them to benefit more from progressive improvements in care.

The age-group interaction (p < 0.001) produced a counterintuitive but clinically important result: the 12–18 year group showed the steepest rate of improvement (β = −0.084%/yr), while the 0–6 year group showed no significant change (β = −0.002%/yr, p = 0.848). This suggests that the secular improvements in glycemic control over the study period were concentrated in school-age and adolescent patients—the age groups most likely to benefit from increased CGM adoption, improved insulin analogues, and structured self-management education (9, 36–38).

The absence of measurable improvement in the youngest age group may reflect several factors. First, a ceiling effect may be present, as younger children often achieve lower baseline HbA1c levels compared to adolescents, leaving limited room for further improvement (39, 40). Second, diabetes management in children under 6 years is entirely caregiver-dependent, which may limit the impact of certain interventions that rely on patient autonomy or self-management skills (21, 39). Third, differential patterns of technology adoption and utilization exist across age groups (37, 41). While CGM use has increased substantially in young children, this age group faces unique challenges including unpredictable eating patterns, variable physical activity, high glycemic variability, and inability to recognize or communicate hypoglycemia, all of which may attenuate the benefits of technological advances (38, 42).

Additionally, glycemic targets have historically been more conservative for very young children due to concerns about severe hypoglycemia and neurodevelopmental risk, though contemporary evidence supports tighter control even in this population (38, 39, 43). Family dynamics and parental involvement patterns also play a critical role in metabolic outcomes, with effects that may supersede those of treatment modality alone (21, 39, 44).

These findings underscore the importance of age-specific interventions in pediatric type 1 diabetes care and warrant further investigation into strategies to optimize glycemic outcomes across all developmental stages.

The plausible contextual contributors to the observed improvement include the 2010 NHI extension of SMBG reimbursement, the 2017 CGM coverage, and the introduction of newer basal insulin analogues from 2016. Evidence from other studies has demonstrated associations between increased SMBG frequency and lower HbA1c (45), and between CGM use and improved glycemic control (46, 47). Our institutional data confirm a substantial increase in CGM adoption (193 sensors in 2020 to 1,208 in 2023) and long-acting insulin analogue use (14.9% in 2018 to 71.4% in 2023). However, because these developments occurred concurrently without a controlled design, their individual contributions cannot be formally isolated, and our findings should be interpreted as describing a population-level secular trend rather than attributing causation to any specific intervention.

The GEE analysis of the binary outcome (HbA1c <7.0%) demonstrated a statistically significant increase in the annual odds of target achievement (OR = 1.029 per year, p = 0.002). The proportion of HbA1c measurements achieving the target increased from 14.1% in 2007 to 28.7% in 2021. This improvement, while clinically meaningful, remains substantially below rates reported in high-technology registries such as Swediabkids (57.1%) or the Czech ČENDA registry (53.6%) (9). The relatively lower insulin pump (CSII) utilization in our cohort—in part due to the absence of NHI reimbursement for insulin pumps—may partially explain this gap. The recent increase in CGM adoption is encouraging and, if sustained and combined with future pump reimbursement, may accelerate target achievement rates.

### Strength and limitation

The primary strength of this study is its 23-year observation span with dense serial HbA1c data from a well-characterized cohort, enabling a rigorous longitudinal analysis using an LME framework with formal interaction testing—an approach not employed in prior Taiwanese studies of this population. The inclusion of diabetes duration as time-varying covariate allows separation of secular, aging, and disease-progression effects on HbA1c.

Several limitations must be acknowledged. First, this study is based on data from two tertiary centers in Taiwan, which limits generalizability to the broader Taiwanese population and to patients managed in primary or community care settings. Second, important time-varying confounders—including insulin pump use, CGM wear time, SMBG frequency, pubertal status, physical activity, and socioeconomic factors—were not available for most of the observation period and could not be incorporated into the model. Third, the LME cannot formally separate calendar-year effects from the concurrent introduction of multiple NHI policy changes; the observed trend represents an aggregate secular change rather than the effect of any individual intervention. Fourth, the HbA1c assay platform was broadly consistent over time, but minor calibration changes over 23 years cannot be entirely excluded. Fifth, the three diagnostic eras serve as a descriptive contextual framework and are not intended to imply that era boundaries precisely define causal transition points. Sixth, the absence of improvement in the 0–6 year group should be interpreted cautiously given the smaller sample size and lower variability in this age stratum.

## Conclusion

Over 23 years, HbA1c levels among children and adolescents with T1D showed a statistically significant declining trend at two MacKay-affiliated centers in Taiwan, concurrent with the progressive adoption of diabetes technology and NHI policy expansion. Formal interaction testing identified significant heterogeneity in improvement rates: female patients and those without DKA at presentation improved more rapidly, and the 12–18 year age group—despite maintaining the highest absolute HbA1c levels—showed the steepest calendar-year decline. The youngest (0–6 year) group showed no significant secular improvement, highlighting a subgroup that may require targeted management strategies. The proportion of HbA1c measurements falling below 7.0% increased significantly per GEE analysis but remains below rates reported in high-technology Western registries, pointing to the continued need for expanded access to insulin pump therapy and sustained NHI support for CGM. Future research should aim to collect comprehensive longitudinal data on technology use, pubertal status, and socioeconomic factors to further elucidate the drivers of glycemic improvement in this population.

Despite limitations in retrospective device tracking, the secular decline in HbA1c levels aligns with the global shift toward precision diabetes management. These improvements occurred in parallel with the progressive institutional adoption of CGM and CSII—contextual developments that are biologically plausible contributors to the observed trend, but whose individual effects cannot be formally isolated within this observational framework. We advocate for the widespread implementation of these technologies as standard pediatric care. In Taiwan, sustained insurance coverage to ensure universal access is paramount for narrowing the gap between clinical targets and real-world outcomes. Furthermore, these observations may provide a valuable framework for improving childhood diabetes care in nations that are at earlier stages of technological progress in this field.

## Data Availability

The original contributions presented in the study are included in the article/**Supplementary Material**. Further inquiries can be directed to the corresponding author.
