# Supplementary material for: Secular trends in HbA1c levels among children and adolescents with type 1 diabetes
Source: Front Endocrinol (Lausanne). 2026 May 28;17:1786897. doi: 10.3389/fendo.2026.1786897 (PMC13253463; doi:10.3389/fendo.2026.1786897)
Supplement: Supplementary file 1 [file Table1.docx]

**Supplementary Material**

**Secular Trends in HbA1c Levels Among Children and Adolescents with Type 1 Diabetes:** A 23-Year Longitudinal Study from Two Centers in Taiwan

**Supplementary Table S1.** Sensitivity analysis: primary calendar-year HbA1c trend across different inclusion thresholds for minimum number of HbA1c measurements

| **Inclusion threshold** | **N patients** | **N observations** | **β/yr** | **SE** | **95% CI** | **p-value** | **τ²** | **ICC** |  |
| --- | --- | --- | --- | --- | --- | --- | --- | --- | --- |
| ≥1 visit | 779 | 24,003 | −0.016 | 0.016 | −0.032, −0.001 | 0.041 | 2.128 | 1.961 | 0.520 |
| ≥2 visits | 742 | 23,966 | −0.026 | 0.008 | −0.041, −0.011 | <0.001 | 1.904 | 1.957 | 0.493 |
| ≥3 visits | 722 | 23,926 | −0.033 | 0.008 | −0.048, −0.018 | <0.001 | 1.845 | 1.949 | 0.486 |
| ≥4 visits | 703 | 23,869 | −0.037 | 0.008 | −0.052, −0.022 | <0.001 | 1.810 | 1.935 | 0.483 |
| **≥5 visits (primary)** | **688** | **23,809** | **−0.037** | **0.008** | **−0.053, −0.021** | **<0.001** | **1.826** | **1.929** | **0.486** |

**Note:** All models use the revised primary specification (Model 2: Age_group only) and include a random intercept per patient (REML estimation), adjusting for diabetes duration at each visit, sex, DKA status at initial presentation, and age group at diagnosis. The primary analysis used a threshold of ≥5 HbA1c measurements, corresponding to at least one year of regular quarterly follow-up per clinical guideline (ADA/ISPAD recommendations for HbA1c measurement every 3 months). Among included patients (≥5 visits), the median interval from first to fifth HbA1c measurement was 12.9 months (IQR 11.5–14.8 months), with 85.9% exceeding 12 months of follow-up. The 91 excluded patients had a median follow-up of 2.8 months; 37 contributed only a single measurement, most likely representing newly diagnosed patients, patients who transferred care, or those with irregular attendance. The calendar-year β is consistent and statistically significant across all thresholds ≥2 visits (range: −0.026 to −0.037%/yr; all p ≤ 0.001), confirming that the primary result is not an artifact of the specific exclusion criterion. The estimate at ≥1 visit attenuates to −0.016%/yr (p = 0.041), likely because single-visit patients contribute no within-patient longitudinal information to the random-intercept model. Primary analysis row is highlighted in yellow. β/yr, estimated HbA1c change per calendar year; SE, standard error; τ², between-patient variance; ICC, intraclass correlation coefficient.
